# Physical activity of first graders in Norwegian after-school programs: A relevant contribution to the development of motor competencies and learning of movements? Investigated utilizing a mixed methods approach

**DOI:** 10.1371/journal.pone.0232486

**Published:** 2020-04-30

**Authors:** Knut Løndal, Anders Lund Hage Haugen, Siv Lund, Kirsti Riiser

**Affiliations:** 1 Department of Primary and Secondary Teacher Education, Faculty of Education and International Studies, Oslo Metropolitan University, Oslo, Norway; 2 Department of Physiotherapy, Faculty of Health Sciences, Oslo Metropolitan University, Oslo, Norway; La Inmaculada Teacher Training Centre (University of Granada), SPAIN

## Abstract

**Background:**

Development of motor competencies and learning of movements in children is dependent on varied physical activity (PA). After-school programs (ASP) might provide opportunities for young schoolchildren to participate in PA. The aim of the current study was to investigate the PA of first graders in ASP and to consider its contribution to the development of motor competencies and the learning of movements.

**Methods:**

The study was performed utilizing a mixed methods design. A total of 42 first graders were sampled from 14 ASPs in Norway. Direct observations of the children’s activities were conducted for the duration of one entire ASP day. PA intensity was measured using ActiGraph accelerometers. Qualitative data were analyzed using qualitative content analysis, while the Mann-Whitney U test and the Wilcoxon signed-rank test were used to analyze the quantitative data.

**Results:**

The median PA time among the observed children was 61.5 minutes. The median stationary time was 75.9 minutes. There was considerable variation within the sample. Girls were significantly more engaged in stationary behavior than boys. Frequent changes in activity type and intensity were typical features of the children’s ASP day. PA duration and intensity were significantly higher outdoors than indoors. Adult-managed time had longer periods of stationary behavior than child-managed time. The PA at all intensity levels contained barrier-breaking movements—especially at light intensity levels.

**Conclusion:**

Most of the first graders studied were engaged in a variety of activity types of different duration and intensity levels, favorable for the development of motor competencies and for the learning of movements. Hence, it is reasonable to highlight that light PA, in combination with moderate and vigorous PA, is also of great importance for children during the time they spend in ASP. Ultimately, there is a need for staff members who can also stimulate varied PA among the most stationary children.

## Introduction

Young schoolchildren in Western societies are living highly institutionalized lives [1, 2] and concerns have been raised about their opportunities for self-determined activity and play [3–5]. Many parents work outside the home and children have to be taken care of by institutions even after school hours [6]. Thus, the responsibility for a significant portion of children’s time after school is assigned to after-school programs (ASP). Consequently, there is need for more knowledge about children’s activities during ASPs and how such institutions can make a positive contribution to children’s lives. Researchers have, from different theoretical perspectives, studied how staying in ASPs influences children’s health, well-being, development, and learning [7–10]. This article focuses on the physical activity of young schoolchildren in ASP.

A widely used definition claims that physical activity (PA) comprises bodily movement that results in energy expenditure and that *type of activity*, *frequency*, *intensity*, and *duration* are its constituting dimensions [11]. According to the terminology consensus published by the *Sedentary Behavior Research Network*, the actions of lying, sitting, and standing are not considered to be PA and should be termed “stationary behavior” [12]. Numerous studies show that there are health and well-being benefits of regular PA in children [13, 14]. Studies also indicate that children’s PA can both contribute to their academic performance and serve as a preventive mechanism against antisocial behavior [15–17]. Evidence-based recommendations establish that children should be physically active at either moderate or vigorous intensity levels (MVPA) for at least 60 minutes per day [18, 19]. A high proportion of the youngest children meet these recommendations but PA significantly decreases as the age of children increases [13, 20]. Although many schoolchildren live physically active lives, there seems to be a small group within this population that is categorized by stationary behavior. This can lead to a vicious circle, ending in sedentary behavior and low PA levels later on in life [21–23].

The importance of children’s *ability* to move with well-adapted movements in play and other activity types is often overlooked as a primary mechanism underlying PA [24]. Previous research suggests that the development of motor competencies during early childhood is an important requirement for PA involvement later on in life [25]. The concept of “motor competence” refers to a person’s ability to perform a variety of motor actions, including coordination of fine and gross motor skills [26]. The correlation between motor competence and physical fitness is shown to be particularly strong among children 4–6 years of age [27]. This may indicate that children with low motor competence are less physically active than their more competent peers. Hence, the relationship between motor competence and PA is both reciprocal and dynamic. Motor competence develops through PA—but PA is also dependent on achieved motor competence. The learning of movements is dependent on a variety of activity types and requires activities that contain a wide range of intensities, from light to vigorous [28–30]. When researchers explain motor competence development and movement learning, they particularly point to the importance of varied experiences in early childhood [31].

Gallahue, Ozmun, and Goodway [28] separate discernible movements into three functional categories: *locomotory movements*, *manipulative movements*, and *stabilizing movements/postures*. Discernible movements might provide a picture of a child’s movement ability. Movements can be located on a continuum—from *basic movements*, through *adaptive movements* and *personal skills and styles*, to *idiosyncratic adjusted movements* [32, 33]. Movements that challenge a child’s previous experiences lead her to change her abilities and to “push” the movements up a continuum—from basic movements toward idiosyncratic adjusted movements [34, 35]. In line with Løndal [8], we define those movements utilized in PA that increase a child’s abilities in this way as *barrier-breaking movements*.

Pellegrini and Smith [36] argue that permanent changes in movement ability can be established without using a goal-oriented approach. Such a holistic view implies that the learning of movements might occur in informal activities and *physical activity play*. Previous research shows that young children acquire well-adapted movements through play behavior and that play activities containing a variety of walking, running, crawling, jumping, skipping, and climbing actions are most common at this age [37].

Schools struggle to provide opportunities for children to meet their daily PA recommendations [38]. Extracurricular PA during recess and ASP time might provide opportunities for children to be more physically active within the school environment [7, 39]. In Norway, ASPs are expected to provide care and supervision for children 6 to 10 years of age, offering them opportunities to engage in play, cultural, and leisure activities [40]. Around 62% of children in grades 1–4 and 81% of first graders (6 years old) attend ASP for 10 to 20 hours each week [41]. Hence, this context offers relevant opportunities to influence PA in children aged 6 to 10.

American studies on how much PA children accumulate while in ASP show that boys exhibit higher levels of MVPA and a lower proportion of sedentary behavior than girls, while older children exhibit less MVPA than younger children [42–45]. However, the average MVPA amount in ASP falls well below half of the recommended 60 minutes per day, which is the formulated goal in several evidence-based recommendations for MVPA in American ASPs [46, 47]. Quantitative and qualitative studies in Norway report that children’s PA during ASP is extensive when time is devoted to child-managed play outdoors [48, 49]. Child-managed activity is characterized by rapid shifts between activity types and intensity levels and consists of a series of short-time activity periods. Although the average PA time is high, some children do not engage in it, which appears to hamper their PA [8].

To our knowledge, no previous empirical studies address both the type and the intensity of PA in ASP while also taking into consideration the value of an activity in relation to the development of motor competencies and the learning of movements. In contrast with sport-dominated extracurricular PA in several other countries [50], Norwegian ASPs are expected to stimulate self-managed activities and play [40]. Hence, a study of children’s PA in this context is of particular interest. Knowledge about modifiable context factors associated with PA, such as outdoor time, type of activity organization, and access to stimulating areas and equipment, is of particular interest for facilitating future interventions tailored to increase valuable PA among children in ASP. Based on arguments intended to prevent the continuance of a sedentary lifestyle, it seems important to discover those children who have a high proportion of stationary behavior and a low level of activity in early childhood [21–23].

The aim of this article is to describe the PA performed by a sample of Norwegian first graders during one day in ASP and to discuss whether and how these children’s activities contribute to the development of motor competencies and the learning of movements. More specifically we investigate:

- The duration and PA intensity of the children’s total ASP time and how these are distributed between outdoor and indoor time, between child-managed and adult-managed time, and between time spent alone and time spent together with others;
- The frequency, duration, and intensity of activity types that the children perform in ASP; and
- The unfolding of the children’s movements while in ASP and whether or not these movements can be described as barrier-breaking.

## Materials and methods

Since the focus of this study is on children’s PA while attending ASP, it was necessary to choose research methods that can provide information about a wide range of PA dimensions. In addition to the quantitative dimensions of intensity, duration, and frequency, we also wanted to obtain information about the qualitative aspects of activities. There are several research methods available for these purposes. Warren et al. [51] provide a research-based overview of the research methods that can be used to explore PA. Based on their evaluation and on our research focus, we chose a combined research design, consisting of *direct observation* and *measurement of PA intensity*. Direct observation can provide both quantitative and qualitative information on PA among children—for instance, the length and frequency of certain activity types (quantitative) and a description of the movements performed during an activity (qualitative). However, direct observation, as a method, is not a very reliable method when seeking to obtain detailed information about the quantitative *PA intensity* dimension. Hence, in line with methodological literature, we utilized objective measurement with accelerometers to achieve this [52].

### Study design

A design that combines quantitative and qualitative methods is conceptualized as *a mixed methods design* [53, 54]. A mixed methods design assigns equal priority to both quantitative and qualitative methods and can provide an opportunity for generating both complementary and deeper understanding of the research issues at hand than either a quantitative or a qualitative approach can do on its own [53]. A mixed methods design is particularly powerful when investigating complex phenomena, processes, and systems. We consider children’s PA to be a complex phenomenon and argue that a mixed methods design can be fruitful for deepening the understanding of this phenomenon. Three levels of integration principles and practices in mixed methods design are highlighted for the purposes of this article, including integration 1) at the study design level, 2) at the methods level, and 3) at the interpretation and reporting level [54]. At the study design level, integration occurred through a convergent design—we collected, in parallel, quantitative and qualitative data and our analysis for integration began after data collection was completed. At the methods level, integration of data occurred through the processes of collecting and merging. First, we linked data through a sampling frame, collecting both quantitative and qualitative data from all children in the sample. Second, data were linked through merging, bringing the datasets together, and conducting the analysis. At the interpretation and reporting level, integration occurred through a narrative, contiguous approach and joint display [54].

### The participating children

We collected information among a subgroup of children who were already participating in a study focusing on first graders’ physical activity play in ASP (*Active Play in ASP*, ClinicalTrials; NCT02954614). The protocol of this study has been previously described [55]. We recruited schools across the municipalities of three counties in the eastern region of Norway. Volunteer school physiotherapists from 14 municipalities assisted in the recruitment of ASPs in schools within their area of responsibility. For the sake of convenience, the schools had to be located less than a 90-minute drive from our research institution. School administrators and employees of 14 ASPs gave their consent to participate. Parents of all first graders attending these ASPs were informed about the study and asked to provide signed consent on behalf of their children—and a total of 456 consented to participate. There were no exclusion criteria. For the present study, we randomly sampled three children from each participating ASP whose parents had provided consent. Both girls and boys had to be represented from each institution. In total, we sampled 20 girls and 22 boys. The data obtained from both the intervention and the control ASPs are analyzed as one sample in the present article.

### Data collection

As part of the intervention study mentioned above, children from 14 ASPs wore ActiGraph GT3X accelerometers (ActigraphTM LLC, Pensacola, US) while attending their ASPs, for a duration of one week, in order to collect objective PA intensity measures. During this week, we observed a subgroup of 42 children over the course of one entire ASP day. In the present study, only accelerometer data from the observation day were included in the analysis. This delimitation was made because accelerometer data and observation data on specific activities had to be analyzed together. Staff members placed accelerometers on each child’s hip at the time of their arrival to the ASP and removed them before they left. Three researchers (ALHH, SL, KL) served as observers and observed 14 children each, one at a time. Altogether, we observed 18 children in October 2016 and 24 children in May 2017, using a pre-prepared observation form that was previously designed for research on PA in Norwegian ASPs [49]. Page one of the form includes textboxes that indicate the beginning and end of each physically active and stationary period, as well as check boxes for predefined activity types, activity places, organizational types, and social contexts. We considered PA to be those activities during which children conducted discernible locomotory movements, manipulative movements, and stabilizing movements/postures [28], as well as activities during which they exhibited stationary behavior, such as lying, sitting, and standing still [12, 20]. The predefined PA types found on the form were walking, running/jumping, climbing, ball activity, activity with other equipment, and sports activities. There were also checkboxes for “other activities” and for “varied play/activities.” When a predefined activity category lasted for at least 20 seconds, we recorded it on the form. Non-predefined activity types that lasted for at least 20 seconds were recorded using a box termed “other activities.” When a child switched between activity types and each type lasted for less than 20 seconds, we used a checkbox termed “varied play/activities.” Page two of the form included a space for qualitative descriptions of each recorded period. We had certain criteria for the types of qualitative aspect we were looking for—what a child actually did during the activity period, how the activity occurred and ceased, exactly where a child was staying during the activity, whether the activity was child-managed or adult-managed, and how social interaction unfolded during the activity. During the observation sessions, we noted qualitative descriptions for each recorded period (see the observation form in Supporting information).

### Pilot study and the inter-rater reliability test

As described in the protocol, we carried out a pilot study among a sample of first graders in two ASPs prior to beginning the data collection [55]. This allowed us to test how accelerometers are worn by six-year-old children and to ensure adequate similarity between the researchers’ coding during the observation sessions. After each observation session, the three observers carefully discussed the observed situations and agreed upon the coding criteria.

In addition, prior to the data collection, we carried out an inter-rater reliability test of the observation form used for the first graders in ASP. The three observers did this by observing the same child simultaneously. First, the same girl was observed for two hours and then the same boy was observed for two hours. Based on the categorizations—recorded on the observation form—we then calculated the Fleiss’ Kappa (K) value to evaluate the inter-rater agreement between the three observers [56]. We were consistent in coding the main PA categories and stationary behavior, respectively (K = 0.62), and in coding how the activities were organized (K = 0.76) as well as where they occurred (K = 1.0). These values reflect substantial (0.61–0.80) and almost perfect agreement (0.81–1.0) [57]. We were less consistent regarding the coding of activity types (K = 0.44) and social contexts (K = 0.26). According to Landis and Koch [57], these values reflect moderate and fair agreement. Aware of these test results—and with a glance at the qualitative descriptions of each period—we discussed specific inequalities in our coding and further agreed upon a unified coding strategy.

### Analysis

KineSoft v3.3.80 (KineSoft, Saskatchewan, Canada) was used for the screening and for the analysis of the accelerometer data. Accelerometer data were stored in 10-second epochs in order to detect the intermittent and sporadic activity patterns of the observed children. PA was defined as all activities (unless they involved stationary behavior), including light, moderate, and vigorous PA. MVPA was estimated with cut points above 2,295 counts per minute (CPM), as described and recommended in previous studies [58, 59].

Distributions of the continuous variables were inspected visually and were deemed to be generally skewed. Additionally, z-values for skewness and kurtosis were calculated for each category of independent variables. Several of these fell outside the range of ±1.96, indicating non-normal distribution of data [60, 61]. Consequently, continuous variables were described with medians and interquartile ranges. The differences between pairs of continuous data were assessed using Mann-Whitney U tests (between subgroups) and Wilcoxon signed-rank tests (intragroup analysis). A significance level of *p* < 0.05 was considered to be statistically significant and all tests were two-sided. After each observation session, we transferred our recordings from the observation form into an Excel file with prepared areas for inputting both quantitative and qualitative information, as well as with pre-prepared formulas for summing up the time and frequency for each predefined quantitative category. We used IBM SPSS version 25 to conduct descriptive statistical analysis of the quantitative data.

We analyzed the qualitative data using qualitative content analysis [62]. The focus in this analysis was placed on the content and on the contextual meaning of the text in order to provide understanding of the children’s movements in PA. According to Braun and Clarke [62], themes and patterns within qualitative data can be identified in either a deductive or an inductive manner, respectively. When we coded our qualitative data, we first took a deductive point of departure. We focused on how the children’s movements during PA periods corresponded with the three functional categories of *locomotory movements*, *manipulative movements*, and *stabilizing movements/postures* [28] and on whether or not the movements seemed to be barrier-breaking [8]. After employing this deductive approach, we followed the steps described by Braun and Clarke [62] in order to perform an inductive search for additional patterns relevant for our research focus. During this process, we revealed some typical patterns that occur during adult-managed versus child-managed PA time.

### Ethical considerations

Prior to conducting the fieldwork, we obtained formal consent from the administration of each participating ASP. We sent a notification about the project to The Norwegian Data Protection Official for Research, who reviewed the study and concluded that the project was in accordance with the Personal Data Act (reference number 46008). We informed the staff members of the ASPs about the study and obtained informed consent from the guardians of the participating children. Prior to the observation sessions, we also obtained verbal consent from the observed children themselves by asking them directly whether or not we were allowed to watch them play, as per the recommendations of Backe-Hansen and Frønes [63]. Furthermore, for the sake of anonymity, the children and staff members mentioned in the examples have been given fictitious names.

## Results

In this section, we present our findings about the physical activity of the 42 participating first graders while in ASP—20 girls and 22 boys. The median age of the children was 6.5 years (range: 1.25 years) on the observation date. The children wore accelerometers during the observation sessions. Unfortunately, one accelerometer failed to store the intensity data during the observation of one participating boy. Thus, while all accelerometer data is available for the 20 observed girls, it is only available for 21 of the 22 observed boys. In total, we coded 6,187.2 minutes (103.1 hours) of individual first graders’ ASP time in 14 Norwegian ASPs. During these coded minutes, we took qualitative field notes related to each of the 1,779 activity/stationary periods recorded. Collectively, the quantitative and the qualitative data give complementary information about the complex phenomenon of the children’s PA. During this presentation, we follow the sequential order in which the research aims were introduced in the introduction. First, we provide overall descriptions of the children’s total ASP time and the activity types that the children performed while in ASP, based on the analysis of quantitative data. Subsequently, we describe the unfolding of the children’s movements in various activities, based on the qualitative analysis.

### Overall descriptions

On average, the observation sessions lasted 147.3 minutes each, reflecting the time that the children spent in ASP on the day on which they were observed.

Table 1 shows how the observed time was distributed between outdoor and indoor time, child-managed and adult-managed time, and time spent alone as well as together with others. Time spent in child-managed activities and time spent together with others were significantly longer than adult-managed and time spent alone, respectively (p < 0.05). As much as 88.6% of the total observed ASP time was spent together with other children and 71.9% of it was child-managed time. PA intensities were significantly higher outdoors than indoors, during child-managed time than during adult-managed time, and during time spent alone than time spent together with others (p < 0.05). However, there was considerable variation within the sample.

**Table 1:** The total observed ASP time, outdoor and indoor time, child- and adult-managed time, and time spent alone as well as together with others, given in median minutes and interquartile range for one ASP day, with the intensity given in median counts per minute (CPM) and interquartile range.

	Total time	Outdoors	Indoors	P-value	Child-managed	Adult-managed	P-value	Alone	Together with others	P-value
**N**	42	42	42		42	42		42	42	
**Median minutes**	146.4	51.5	70.8	0.088	106.9	34.2	0.000*	11.2	126.3	0.000*
**Interquartile range**	104.5	75.0	80.3		80.3	36.9		20.8	109.4	
**N**	41	41	41		41	41		41	41	
**Median CPM**	1106	1536	607	0.000*	1337	701	0.000*	1583	1025	0.003*
**Interquartile range**	690	668	328		814	648		891	727	

* = statistically significant

The children were physically active 45.1% of the total observed ASP time and they were engaged in stationary behavior (lying, sitting, and standing still) 54.9% of the time. The median PA time and the stationary behavior time were 61.5 and 75.9 minutes, respectively (see Table 2).

**Table 2:** The total time, PA time, and stationary behavior time (SB) in ASP, given in median minutes and interquartile range for one observed ASP day, with the intensity given in median CPM and interquartile range. P-values are given for the difference between subgroups.

		Minutes in observed ASP time				Intensity (CPM) during ASP time			
		All (n = 42)	Girls (n = 20)	Boys (n = 22)	P-value	All (n = 41)	Girls (n = 20)	Boys (n = 21)	P-value
**Total**	**Median**	146.4	161.5	130.3	0.199	1106	1080	1224	0.167
	**Interquartile range**	104.5	106.5	106.6		690	627	928	
**PA time**	**Median**	61.5	52.8	66.1	0.290	1863	1864	1863	0.774
	**Interquartile range**	60.9	70.1	53.3		775	869	774	
**SB time**	**Median**	75.9	93.2	57.2	0.016*	511	549	510	0.531
	**Interquartile range**	62.0	41.5	54.0		357	317	320	

* = statistically significant

The findings show that the girls were significantly more engaged in stationary behavior than the boys (p < 0.05). On average, they spent about as much time in PA as boys but the PA percentage of their total ASP time was less than that of the boys. The Mann-Whitney U tests did not reveal significant intensity differences (CPM) between girls and boys during either physically active or stationary time.

### Physical activity types

We collected qualitative information about both physically active periods and stationary behavior periods. In this paper, we particularly focused on the physically active periods that unfolded. We plan to describe and discuss the results from the stationary periods in an upcoming article in more detail. Table 3 shows how the time spent in physical activity was distributed between various activity types, how the median minutes were spent, and what the intensity level for each activity type was.

**Table 3:** The activity types given in total, median physically active minutes for the observed ASP time, and the intensity given in median CPM among the children performing each activity type; N = total number of observed children; n = number of children observed in the activity type.

	Total PA time	Outdoors PA time	Indoors PA time	P-value	Varied PA	Walking	Running / Jumping	Climbing	Ball-activities	PA, other equipment	Tricycles and scooters	Other PA
**N**	42	42	42		42	42	42	42	42	42	42	42
**n**	42	34	42		29	42	28	17	18	29	9	17
**Total min.**	2794.3	1866.2	928.1		570.5	694.8	136.7	95.5	439.5	569.4	129.7	158.5
**Median min.**	62.0	46.1	9.3	0.018*	7.8	13.7	1.4	0.0	0.0	10.4	0.0	0.0
**Interquartile range**	60.9	55.0	32.8		20.3	15.9	4.5	2.5	19.1	20.0	0.0	3.2
**N**	41	41	41		41	41	41	41	41	41	41	41
**n**	41	33	41		29	41	27	16	17	28	9	16
**Median CPM**	1863	1862	1313	0,000*	1776	1365	2261	1688	2418	1957	1139	2284
**Interquartile range**	775	779	901		1005	747	1050	752	1019	1420	1135	1524

* = statistically significant

The findings show that PA duration and intensity were significantly higher outdoors than indoors (p < 0.05). A typical feature of first grader PA during ASP time was frequent change of activity types. We noticed that the children were engaged in PA with loose or moveable equipment for the majority of the ASP time (40.7% of the total observed PA time)—*ball activities*, *PA with other equipment*, and *PA with tricycles and scooters*. *Ball activities*, *PA with other equipment*, and *other PA*—together with *running/jumping*—were characterized by higher intensity levels than other activity types. These activity types had median intensity levels above or near the MVPA cut. *Other PA* comprised mainly dance, balancing games, and rough and tumble play. It is worth noting that the median intensity levels for *PA with tricycles and scooters*, *climbing*, *walking*, and *varied PA* were well below the cut point set for MVPA.

### The unfolding of the children’s movements while in ASP

The qualitative content analysis revealed some typical patterns in adult-managed versus child-managed time. Adult-managed time was usually organized in groups of children and mainly included stationary activities that took place indoors. Much of the adult-managed time consisted of facilitating meals for all children, although there were also examples of organized groups within varied arts and crafts activities (e.g., drawing, face painting, sewing) and some examples of organized PA (e.g., ballgames). All adult-managed activities, except for meals, were voluntary. In one situation during an observation session, described below, a young man (Jack) organized a voluntary PA session in the school’s gym hall.

Ethan and the other kids are sitting in a circle marked on the gym hall floor. Jack explains the warm up game in detail: groups of five kids shall line up one behind the other and run in a row; the front-runner shall perform movements that the others shall imitate. The game starts and unfolds with high intensity and Ethan gets one period as front-runner. After a few minutes, the kids have to sit down in the circle again. Jack has planned for a game of indoor soccer. He explains the rules carefully and the kids remain sitting for several minutes. Finally, the kids are placed in two teams and the game begins. Jack often stops the play in order to explain rules. The game lasts for seven minutes and then the kids have to sit down in the circle again. Jack introduces a finishing game, a competition in which the loser of each session has to leave the game. The activity is vigorous but Ethan is “knocked out” in the first round. Thereafter, he stands still, waiting until the winner is crowned.

This was a typical ASP situation observed in terms of adult-managed physical activities, both indoors and outdoors, which often included long periods of stationary behavior. Child-managed physical activities commonly appeared in the form of physical activity play outdoors. These activities also included stationary periods but the durations of these periods were usually short. Child-managed time, particularly outdoors, had frequent changes in intensity levels and between activity types containing a variety of locomotory movements, manipulative movements, and stabilizing movements/postures. The average duration of the PA periods recorded during all observation sessions was 2.9 minutes. The combination of quantitative and qualitative data collected through observations and objective PA intensity measurements provided us with an opportunity to also consider qualities in children’s physical activities other than intensity and duration. The results of the qualitative thematic content analysis, in particular, shed light on periods of light PA that include barrier-breaking movements. During the observations, we recorded many such periods. Below, we describe two such situations and relate them to PA intensity level. In the first situation, the focus is on Megan—who challenges her balance skills together with two other girls. In the second situation, we focus on Thom—who performs fine and gross motor movements in the sandpit.

Megan and two other girls play on a balance beam that is located between some trees in the outdoor area and a balancing game occurs. The balance beam consists of a horizontal timber log that is mounted so it can rotate around the longitudinal axis. There is a rope line installed to hold onto while balancing. The girls stand on the beam simultaneously and they hold onto the rope. They are singing and laughing while Megan makes squats balancing on the beam. The other two girls move the rope up and down to make the movement more difficult for Megan. The girls change roles and continue the balancing game for about 26 minutes.

The balancing game appears to be very challenging and the girls carry out stabilizing movements and postures that seem to be at the limit of their motor competencies. The objective measurements of Megan’s PA intensity during the 26.2 minutes of the balance game show an average of 565 CPM, far below the MVPA level.

Thom plays in the sandpit together with one other boy. They use spades and other digging equipment and are playing at a slow pace. They alternate between digging and building structures in the sand. Thom plays mostly alone during this period but he often speaks to his friend. He seems to be immersed in shaping and molding of structures in the sand. Among other things, he makes a tunnel that he manages to send a stone through. When his friend has to go home, Thom continues his digging and shaping activity.

We also checked the PA intensity level for Thom’s immersed motor activity containing manipulative movements in the sandpit. The average PA intensity level is light during this 43.2-minute long period, at 1,482 CPM.

## Discussion

Until now, little has been known about young schoolchildren’s PA while in ASP, especially about what the children actually do during PA time, how the activities occur, unfold, and cease, where the children are staying during PA, whether the activities are child- or adult-managed, and how social interaction unfolds during these activities. The results presented above reveal some interesting issues for discussion, especially in terms of how child-managed outdoor time contributes to PA, how adult-managed time contributes to stationary behavior, and how light PA might contribute to the development and learning of motor competencies.

Our study shows that, on average, the 42 observed first graders were physically active through locomotory movements, manipulative movements, or stabilizing movements/postures for 45.1% of the observed ASP time and that much of this activity occurred during self-chosen and child-managed activities in physical activity play. Outdoor time contributed to the children’s physical activity more than indoor time did. This applies to both the total PA time and the intensity level. In addition, there was generally less stationary behavior outdoors than indoors. These findings are in line with previous research on children’s PA in ASP [45, 49]. However, the results of our study show large variations between the observed children. The results published in a recent article from the *Active Play in ASP* project indicate that both light PA and MVPA increase in activities that occur outdoors in comparison with those that occur indoors for both the most and the least active children [48]. Based on these results, including mandatory outdoor time during each ASP day should be considered. This appears to be a relevant organizational approach that can contribute to more PA and less stationary behavior in children—even the least active ones.

The observed children participated in adult-managed activities for only 28.1% of ASP time. It is worth noting that most of the adult-managed activities took place in organized child groups indoors and consisted of stationary behavior. Most of these activities can be considered to be positive elements in an ASP context—among other things, they contribute to close adult-child contact and deep adult-child conversations. However, we should reflect on whether it is desirable for most adult-managed activities to include voluntary, stationary activities indoors and, as described in the example with Ethan presented in the results section, for adult-managed PA to be characterized by long periods of stationary behavior. It is particularly interesting to discuss this in light of the large variation in PA found among the children who participated in the study. A recent study on extracurricular activity among Canadian fourth graders also showed great variations in the children’s activity levels, revealing that the children who engaged in PA and sport activities together with others scored higher on measurements related to well-being and health than non-active children [64]. Intervention studies in American ASPs indicate that structured after-school PA programing might increase the children’s activity levels [65]. Hence, there seems to be a potential for ASP staff to contribute to an increase in PA among the least active children. In line with previous research on ASPs [42–45], our study shows that boys are more physically active than girls and that girls are more engaged in stationary behavior than boys. Therefore, there is reason to ask whether adult-managed, stationary activities facilitated in ASPs are generally more attractive to girls than to boys and whether the staff should focus more on facilitating physical activity play for girls. However, to determine which children need particular support to become involved in PA, to facilitate varied and tailored types of activities that can stimulate motor competence, and to contribute to including all children in physically active play requires professional competence. There seems to be a need for staff members who can determine, to a higher extent than is currently the case, which children need particular support to become involved in PA. There is also a need for competence in facilitating varied and tailored types of activities that can stimulate motor competencies. This is a deep pedagogical competence that requires both formal education and reflected practical experience. In Norway, there is no pedagogical education requirement for ASP staff members. Hence, there is a need for the development of training programs that are adapted for ASP staff.

As mentioned previously in this article, Gallahue et al. [28] distinguish between three main categories of discernible movements—locomotory movements, manipulative movements, and stabilizing movements/postures—and these categories are highlighted as important for the development of motor competencies and the learning of movements. The results from our study indicate that self-chosen and child-managed activities, with loose and moveable equipment, are particularly PA promoting. As shown in the balancing game and sandpit examples described in the results section, such activities require advanced stabilizing movements/postures and manipulative movements. In our material, these movements appear in the form of balancing games, climbing activities, ball activities, activities with different types of bicycles, and activities with equipment such as hula hoops, digging equipment, jumping ropes, etc. In total, such manipulative movements represented over 40% of the total PA during ASP time among the observed first graders. This finding points to the importance of having suitable equipment and play installations available for the relevant age group and of making this equipment available during ASP time. Such facilitation of suitable environment is in line with the dynamic system theory [32, 33]—where the development of motor competencies is dependent of the interaction between the child and her environment. The other two main categories of discernible movements—locomotory movements and stabilizing movements/postures [28]—are also strongly represented in our observation material, most often in the form of physical activity play.

The relatively short portion of the day (median 146.4 minutes) during which first graders attend ASP contributes to a considerable amount of PA that consists of varied types and intensity levels (median 61.5 minutes). Much of this activity is light PA and, hence, falls under the intensity levels (MVPA) that health recommendations highlight [18]. Previously published results from the *Active Play in ASP* project show that the participating first graders accumulated, on average, 25.8 minutes of MVPA during their ASP stay [48]. Taking ASP time into account, this is a considerable portion of the recommended 60 daily minutes of MVPA. Furthermore, when we take the development of motor competencies into consideration, light PA is also found to be of great importance. Light PA are typically seen in activities developing gross motor skills and object-control skills in children [28–30]. Development of motor competencies is dependent on a variety of movement and activity types and requires activities that contain a wide range of intensity, from light to vigorous. The results of our study show a frequent variation in activity types and intensity levels. They also indicate that light PA often involves what Løndal [8] describes as barrier-breaking movements—locomotory movements, manipulative movements, and stabilizing movements/postures at the limit of the children’s motor competencies. Light PA constitutes movements/postures that are suitable for “pushing” the child’s movements up the continuum from basic movements toward idiosyncratic ones [28]. Therefore, when assessing children’s PA, MVPA should not solely be focused upon—as light PA involving barrier-breaking movements seems to play an important role in children’s motor development and learning.

### Strengths and limitations

The mixed methods design of our study allowed us to address both the quantitative and qualitative aspects of children’s PA during ASP time while simultaneously taking PA intensity into consideration. We consider this to be a strength of the study. We also consider it a strength that we used recognized instruments to conduct objective PA intensity measurements. Nevertheless, there are also certain limitations. Three researchers conducted observations, which may have led to somewhat different recording of activity types. We attempted to solve this problem through an inter-rater agreement test for the observation form used in the study. It is our impression that the discussions during the testing process improved the quality of the coding. Additionally, the results of the inter-rater agreement test made us conscious of the importance of additional qualitative descriptions for each recorded PA period. This constitutes an important argument for using a mixed methods design in studies that focus on PA among children.

## Conclusion

Most of the first graders are physically active for a high proportion of the time they spend in ASP and they are engaged in a variety of movement and activity types, with different intensity levels and durations, most often during physical activity play. The frequent changes between activity types and intensity levels are typical features observed during children’s ASP time. The results show that PA at all intensity levels contains barrier-breaking movements favorable for the development of motor competencies and the learning of movements, particularly at light intensity levels. It seems that PA intensity often has to be decreased when children engage in challenging barrier-breaking manipulative movements and stabilizing movements/postures. Hence, there is reason to highlight that light PA, in combination with activities within MVPA, is of great importance during the children’s ASP time.

This study revealed that most of the first graders are physically active during self-chosen physical activity play and that the activities promote the development of motor competencies and the learning of movements. It also revealed a tendency for adult-managed time to contribute to the children’s stationary behavior. The findings should influence future ASP practices and the education of ASP staff. In general, it seems important to place emphasis on facilitating child-managed play, particularly outdoors. This requires suitable equipment and play installations to be made available to the relevant age group and for this equipment to be available during ASP time. Additionally, emphasis should be placed on opportunities given to staff to discover and support the least active children to become involved in PA. Competence in facilitating physical activity play and in supporting PA among the children who are most engaged in stationary behavior is a pedagogical competence that should be highlighted in future ASP staff education.

## Supporting information

S1 Data Observation form translated from Norwegian to English. (PDF)
