# Supplementary material for: Physical activity of first graders in Norwegian after-school programs: A relevant contribution to the development of motor competencies and learning of movements? Investigated utilizing a mixed methods approach
Source: PLoS One. 2020 Apr 30;15(4):e0232486. doi: 10.1371/journal.pone.0232486 (PMC7192409; doi:10.1371/journal.pone.0232486)
Supplement: S1 Data — (PDF) [file pone.0232486.s001.pdf]

OBSERVATION SCHEME – PHYSICAL ACTIVITY PLAY IN ASP

Child:		ACTIVITY TYPE											PLACE						MANAGEMENT				SOCIAL SETTING		
School:		INACTIVE			PHYSICAL ACTIVE								OUTDOOR			INDOOR			CHILD-MANAGED		ADULT-MANAGED				
	TIME	SITTING / LYING	SITTING WITH SMALL MOVEMENTS	STANDING	VARIED PLAY / ACTIVITY	WALKING	RUNNING/JUMPING	CLIMBING	ACTIVITIES WITH BALL	AKTIVITIES WITH OTHER EQUIPMENT	SPORT ACTIVITIES	OTHER ACTIVITIES	SCHOOL PLAYGROUND	NATURE AREA	OTHER PLACE	ASP AREA	OTHER PLACE AT SCHOOL	OTHER PLACE	SELFCHOSEN AND CHILD- MANAGED	CHOSEN FOM A MENU	ASP STAFF	OTHER THEACHER/ INSTRUCTOR	ALONE	TOGETHER WITH ONE	PART OF A GROUPE
1																									
2																									
3																									
4																									
5																									
6																									
7																									
8																									
9																									
10																									
11																									
12																									
13																									
14																									
15																									

Comment on each activity period

Nr.	Comment:
1	
2	
3	
4	
5	
6	
7	
8	
9	
10	
11	
12	
13	
14	
15	
